# Triterpenoids from the Leaves of *Centella asiatica* Inhibit Ionizing Radiation-Induced Migration and Invasion of Human Lung Cancer Cells

**DOI:** 10.1155/2020/3683460

**Published:** 2020-09-15

**Authors:** Ah-Reum Han, Sanghun Lee, Sujin Han, Yeon Jin Lee, Jin-Baek Kim, Eun Kyoung Seo, Chan-Hun Jung

**Affiliations:** ^1^Advanced Radiation Technology Institute, Korea Atomic Energy Research Institute, Jeongeup-si, Jeollabuk-do 56212, Republic of Korea; ^2^Jaseng Spine and Joint Research Institute, Jaseng Medical Foundation, Seoul 06110, Republic of Korea; ^3^College of Pharmacy, Graduate School of Pharmaceutical Sciences, Ewha Womans University, Seoul 03760, Republic of Korea; ^4^Jeonju AgroBio-Materials Institute, Jeonju-si, Jeollabuk-do 54810, Republic of Korea

## Abstract

Radiotherapy using ionizing radiation is a major therapeutic modality for advanced human lung cancers. However, ionizing radiation itself can induce malignant behaviors such as cancer cell migration and invasion, leading to local recurrence or distal metastasis. Therefore, safer and more effective agents that inhibit the metastatic behaviors of cancer cells in radiotherapy are needed. As a part of our ongoing search for new radiotherapy enhancers from medicinal herbs, we isolated the following triterpenoids from the ethanol extract of C*entella asiatica*: asiatic acid (1), madecassic acid (2), and asiaticoside (3). These compounds inhibited the ionizing radiation-induced migration and invasion of A549 human lung cancer cells at noncytotoxic concentrations. These results suggest that triterpenoids 1–3 isolated from *C*. *asiatica* are candidate natural compounds to enhance the effect of radiotherapy in patients with non-small-cell lung cancer.

## 1. Introduction

*Centella asiatica* (family Apiaceae), commonly known as Indian Pennywort, is an ethnomedical plant that is widely used in India for treating skin problems and for revitalizing the brain and nervous system [1]. It has been reported to have various pharmacological activities, including antioxidant, anti-inflammatory, anticancer, neuroprotective, cardioprotective, skin protective, radioprotective, immunomodulatory, memory-enhancing, and wound-healing properties [2, 3]. Studies on the chemical constituents of *C. asiatica* showed the presence of pentacyclic triterpenoids known as centelloids. These compounds are composed of terpene acids along with glycosides, including asiatic acid, asiaticoside, madecassic acid, madecassoside, brahmic acid, brahmoside, brahminoside, thankiniside, isothankunisode, madasiatic acid, centic acid, centelloside, and cenellic acid [4, 5]. Asiatic acid and its derivatives possess a broad spectrum of pharmacological activities, such as anticancer [6–8], wound healing [9, 10], anti-inflammatory [10, 11], antidiabetic [12], hepatoprotective [13, 14], and neuroprotective activities [15, 16]. In addition, *C. asiatica* contains flavonoids, phenolic acids, and volatile oils, which exhibit antioxidant activity [17–19].

Lung cancer is the most common type of cancer and is associated with a high rate of cancer-related mortality worldwide [20]. Lung cancer is generally divided into two major subtypes: non-small-cell lung cancer (NSCLC) and small-cell lung cancer, accounting for 85% and 15% of all lung cancers, respectively [21]. More than 70% of patients with NSCLC are diagnosed at an advanced stage with metastatic disease (stage III or IV) [22]. Current treatment options for lung cancer include surgical resection, chemotherapy, and radiotherapy. However, for inoperable stage III and IV NSCLCs, radiotherapy and chemotherapy are considered the standard therapy [23]. Radiotherapy involves exposure to ionizing radiation (IR) to either directly or indirectly kill cancer cells, with the main advantage of localized application in most cases. However, IR can also promote malignant effects in some cases, such as local recurrence and distant metastasis [24]. IR at sublethal doses has been shown to promote cancer cell migration and invasion in various cancer cell lines [25, 26], and it induced metastasis in an animal model [27]. Furthermore, IR induces radioresistance, which contributes to the poor prognosis of patients with cancer, and it is the main reason for radiotherapy failure [28]. These effects can lead to tumor recurrence and metastases. Therefore, it is necessary to develop an effective and safe radiotherapy enhancer that can inhibit the induction of malignant behavior.

Although several studies have shown the anticancer effects of extracts and constituents from *C. asiatica* [6–8, 29], there has been no investigation on the inhibitory effects of *C. asiatica* components on IR-induced migration and invasion of cancer cells. Therefore, as a part of our ongoing search for new radiotherapy enhancers from medicinal herbs, we isolated three triterpenoids from the ethanol extract of *C. asiatica* and evaluated their inhibitory activities on the IR-induced migration and invasion of A549 human lung cancer cells.

## 2. Materials and Methods

### 2.1. Extraction and Isolation of Compounds from *C. asiatica*

The ethanol extract of *C. asiatica* (20 g; InterPharm Co., Seoul, Korea) was separated by silica gel thin-layer column chromatography (CC) using CHCl_3_–MeOH (1 : 0 to 0 : 1, v/v) as a gradient solvent system with an RP-18 column (YMC gel ODS-A, 12 nm, S-150 *μ*m; YMC Co., Japan), yielding fractions F1–F10. Fraction F2 (3 g) was further subjected to silica gel CC using hexane-acetone (1 : 0 to 0 : 1, v/v) to afford subfractions F2.1–F2.7. Fraction F2.2 (1.2 g) was then subjected to reversed-phase CC with MeOH-H_2_O (1 : 1 to 0 : 1, v/v) as a solvent system to yield compound 1 (532 mg). Fraction F2.5 (0.8 g) was purified by reversed-phase CC with CH_3_CN–H_2_O (3 : 2 to 1 : 0, v/v), resulting in the isolation of compound 2 (258 mg). Fractions F8 and F9 (3.2 g) were combined and subjected to silica gel CC using gradient mixtures of CHCl_3_–MeOH–H_2_O (7 : 3:0.3 to 6 : 4:0.5, v/v/v) to obtain subfractions F8.1–F8.7. Fraction F8.5 (1.0 g) was chromatographed over ODS-A (120 g) using an isocratic solvent system of MeOH-H_2_O (7 : 3, v/v) to yield compound 3 (351 mg). Thin-layer chromatographic analysis was performed on the Kieselgel 60 F_254_ (silica gel, 230–400 mesh, 0.25 mm layer thickness; Merck, Germany) and RP-18 F_254s_ (Merck) plates, visualized under ultraviolet light (254 and 365 nm) and 10% (v/v) sulfuric acid spray, and then heated (120°C, 5 min). The compounds were identified by one- and two-dimensional nuclear magnetic spectra recorded on a UNITY INOVA 400 MHz FT-NMR instrument with tetramethylsilane as the internal standard. Optical rotations were measured on a JASCO P-2000 polarimeter.

### 2.2. Cell Culture

Human NSCLC cell line A549 was purchased from the Korean Cell Line Bank (Seoul, Korea). The cells were cultured in RPMI-1640 medium (Hyclone, Logan, UT, USA) supplemented with 10% fetal bovine serum (FBS; Hyclone) at 37°C in a 5% CO_2_ incubator.

### 2.3. Cell Viability Assay

The Cell Counting Kit-8 (CCK-8) assay kit (Dojindo, Kumamoto, Japan) was used to evaluate cell viability according to the manufacturer's protocols. In brief, the cells were seeded in 96-well plates at a density of 2 × 10^3^ cells/well and incubated at 37°C for 24 h. The cultured cells were then treated with each compound at various concentrations (0.78–200 *μ*M). After 48 and 72 h of incubation, 10 *μ*L of CCK-8 reagent was added to the cultured cells, which were then incubated for another 4 h; the absorbance of the sample was measured at 450 nm on a VICTOR3 1420 Multilabel Counter (PerkinElmer, Waltham, MA, USA). The 50% inhibitory concentration (IC_50_) was calculated from the dose-response curve using GraphPad software (GraphPad Software, La Jolla, CA, USA).

### 2.4. Wound-Healing Assay

Cell migration potential was evaluated using a wound-healing assay, as described previously [30]. In brief, the cells were seeded in a 24-well plate (3.5 × 10^5^ cells/well) containing plastic inserts (Cell Biolabs Inc., San Diego, CA, USA) for the generation of a wound field and incubated for 24 h. After removing the inserts from the wells, the cells were exposed to 10 Gy *γ*-irradiation using a ^137^Cs *γ*-ray source (Atomic Energy of Canada, Mississauga, ON, Canada) at a dose rate of 3 Gy/min. The irradiated cells were incubated with each compound at various concentrations (2.5, 5, and 50 *μ*M). Images were analyzed at 0 and 24 h using an AE31 microscope (Motic, Hong Kong).

### 2.5. Invasion Assay

Cell invasion potential was assessed using a transwell assay, as described previously [31]. In brief, IR-treated (10 Gy) or nontreated (0 Gy) A549 cells in serum-free medium were seeded (1 × 10^4^ cells) on the upper surface of Matrigel-coated transwell chambers (BD Biosciences). The lower compartments of the chambers were filled with medium supplemented with 10% FBS. After 16 h of incubation, the cells that invaded the lower surface of the filter were stained using the Diff-Quick Kit (Fisher Scientific) and counted under a microscope (Motic).

### 2.6. Statistical Analysis

All experiments were repeated at least three times. Statistical significance was determined using Student's *t*-test or one-way analysis of variance (GraphPad Software).

## 3. Results and Discussion

### 3.1. Isolation and Identification of Triterpenoids from the Ethanol Extract of *C. asiatica*

Medicinal herbs are a rich source of various bioactive compounds, which have long been used in the treatment of many diseases, including cancer [32]. Recently, compounds isolated from medicinal herbs have been identified as potent radiotherapy enhancers, such as radiosensitizers [33]. In this context, we have been searching for novel potent radiotherapy enhancers against IR-induced cancer cell migration and invasion from *C. asiatica*.

**Figure 1:**
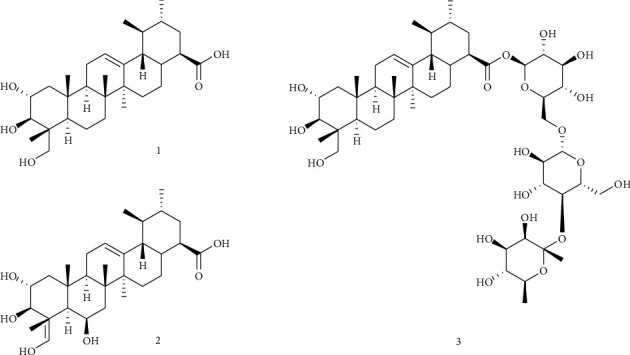
Chemical structure of compounds 1–3 isolated from *Centella asiatica*.

By chromatographic separation of the ethanol extract of *C. asiatica*, we isolated three compounds, which were identified as asiatic acid (1) [34], asiaticoside (3) [35], and madecassic acid (2) [36], by NMR spectra analysis and comparison with their published values (Figure 1).

Asiatic acid (1), asiaticoside (2), and madecassic acid (3) have been reported as the major components of *C. asiatica* [34–36], and these compounds have exhibited anticancer activity in various cancer cell lines. Asiatic acid has showed an antiproliferative effect by regulating apoptosis in a variety of human cancer cells, such as breast cancer, lung cancer, and melanoma cells [6]. Madecassic acid has also been found to inhibit cell growth by inducing apoptosis in mouse colon cancer cells and exhibit antiproliferative activities in various cancer cell lines via regulation of the ERK signaling pathway [7]. A recent study showed that the anticancer effect of asiaticoside is mediated by the inhibition of cell migration and invasion via the STAT3 signaling pathway in multiple myeloma [8]. However, the anticancer activity of these compounds in the context of their potential as radiotherapy enhancers has not been assessed.

### 3.2. Inhibitory Activities of Compounds 1–3 on IR-Induced Migration in A549 Cells

**Figure 2:**
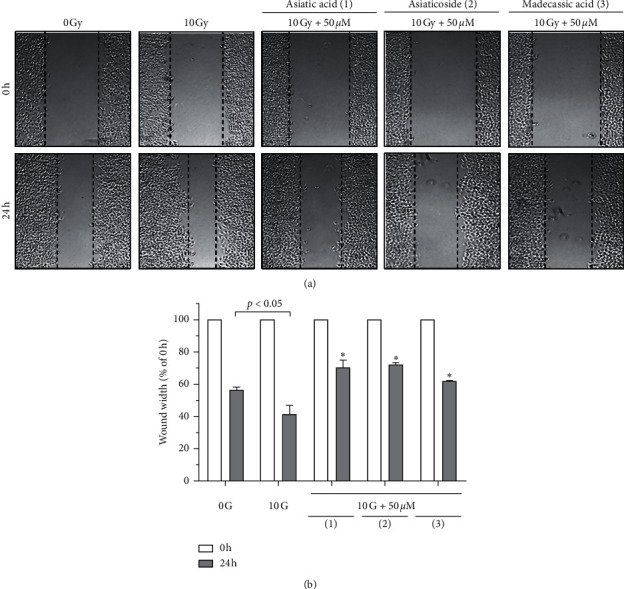
Effects of compounds 1–3 on IR-induced A549 cell migration: (a) wound-healing assay to examine the effects of the indicated compounds (50 *μ*M) on the IR-induced migration of A549 cells; (b) quantification of the wound width. The relative wound width was calculated as the ratio of the remaining wound width at a given time point to the original wound width created at 0 h Data represent the mean ± SD (*n* = 3),^*∗*^*p* < 0.005 versus the control (10 Gy IR).

To search for potent radiotherapy enhancers from medicinal herbs, we screened their constituents at a concentration of 50 *μ*M to evaluate the inhibitory effects on IR-induced A549 cell migration [37].  Figure 2(a) shows the width of the scratch created, which mimics a wound, that was rapidly covered by cells following 10 Gy *γ*-irradiation. However, treatment with 50 *μ*M of compounds 1–3 decreased the covering rate over the wound area. To quantify cell migration, the width of the scratch created in A549 cells was measured and calculated at 0 and 24 h after wound creation. As shown in Figure 2(b), all tested compounds significantly suppressed the wound-healing ability of *γ*-irradiated A549 cells in comparison with the untreated *γ*-irradiated A549 cells. This finding indicated that compounds 1–3 inhibited IR-induced A549 cell migration.

### 3.3. Cytotoxicity of Compounds 1–3

**Figure 3:**
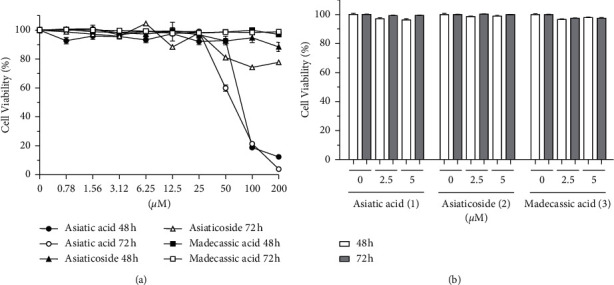
Effect of compounds 1–3 on the viability of A549 cells. Viability of A549 cells (2 × 10^3^ cells/well) was estimated using the CCK-8 assay after treatment with the indicated compounds at (a) 0.78–200 *μ*M and (b) 2.5 and 5 *μ*M for 48 and 72 h. The values are expressed as mean ± SD of three independent experiments.

To investigate whether the inhibitory effects on IR-induced A549 cell migration were actually due to cytotoxicity, the cell viability assay was performed. In this study, the effect of 10 Gy IR on the viability of A549 cells was not tested because it has been reported to have no effect on the viability in A539 cells [38]. The results of the CCK-8 assay demonstrated that compound 1 (IC_50_ value at 48 and 72 *h* = 69.94 and 57.40 *μ*M, respectively) exhibited cytotoxic effects at 50 *μ*M, whereas compounds 2–3 did not (IC_50_ values at 48 and 72 *h* =>200 *μ*M) (Figure 3(a)). These results suggested that the antimigratory activity of compound 1 on *γ*-irradiated A549 cells can be partially attributed to its cytotoxicity, whereas compounds 2–3 appear to have inhibitory effects that are independent of cytotoxicity. Next, to determine the noncytotoxic concentration of 1–3, the cell viability assay was performed with a low concentration of each compound (2.5 or 5 *μ*M). As shown in Figure 3(b), compounds 1–3 did not exhibit cytotoxic effects at these concentrations; thus, further analyses were performed at these concentrations.

### 3.4. Inhibition of IR-Induced Migration in A549 Cells by Compounds 1–3

**Figure 4:**
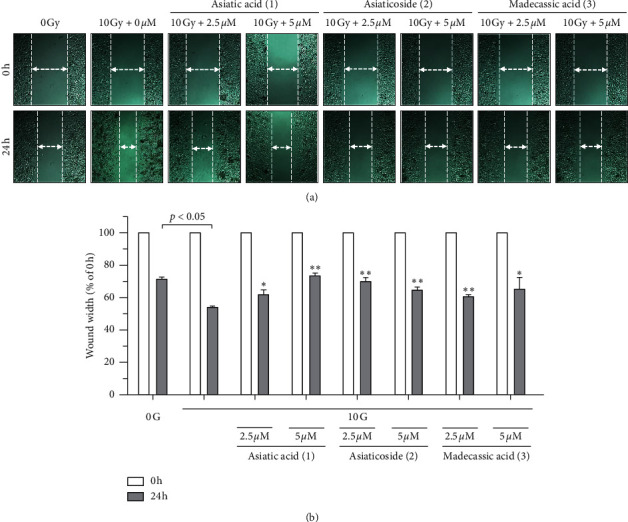
Effects of compounds 1–3 on IR-induced A549 cell migration: (a) wound-healing assay to examine the effects of the indicated compounds (2.5 and 5 *μ*M) on the IR-induced migration of A549 cells; (b) quantification of the wound width. The relative wound width was calculated as the ratio of the remaining wound width at the given time point to that of the original wound width created at 0 h. Data represent the mean ± SD (*n* = 3); ^*∗*^*p* < 0.05; ^*∗∗*^*p* < 0.005 versus the control (10 Gy IR).

The wound-healing assay was performed by treating the cells at the noncytotoxic concentrations of compounds 1–3 (2.5 or 5 *μ*M) and then exposing to 10 Gy IR. As shown in Figure 4, compounds 1–3 decreased the covering rate of the width of the scratch created in a dose-dependent manner, which was confirmed by quantification of cell migration. These findings indicated that compounds 1–3 can inhibit IR-induced A549 cell migration at a noncytotoxic concentration.

### 3.5. Inhibition of IR-Induced Invasion in A549 Cells by Compounds 1–3

**Figure 5:**
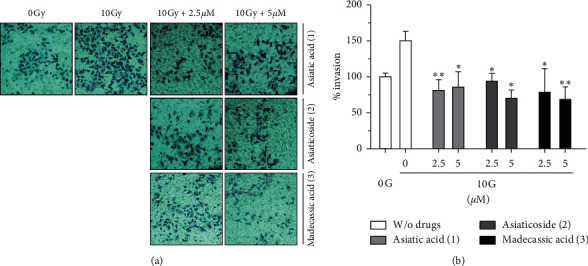
Effects of compounds 1–3 on the IR-induced invasion of A549 cells: (a) cell invasion was assessed using Matrigel-coated transwell plates in nonirradiated and *γ*-irradiated A549 cells treated with 2.5 and 5 *μ*M compounds 1–3, respectively; (b) quantification of invasion. The percentage of invasion is represented as the number of cells per field compared with each nonirradiated A549 cell group. Data represent the mean ± SD (*n* = 3); ^*∗*^*p* < 0.05; ^*∗∗*^*p* < 0.005 versus the control.

The transwell assay showed that the invasion of A549 cells was induced by 10 Gy IR, and it considerably decreased after treatment with compounds 1–3 (Figure 5(a)). All tested compounds (at the noncytotoxic concentrations) significantly suppressed the invasiveness of *γ*-irradiated A549 cells in comparison with that of the untreated *γ*-irradiated A549 cells (Figure 5(b)). These results suggested that compounds 1–3 inhibited IR-induced A549 cell invasion at noncytotoxic concentrations.

## 4. Conclusions

In conclusion, we demonstrated that compounds 1–3 isolated from *C. asiatica* with demonstrated anticancer activity can also inhibit the IR-induced migration and invasion of A549 lung cancer cells. These findings suggest that compounds 1–3 might be effective in improving the radiotherapeutic effect in NSCLC. However, further research on the inhibitory mechanisms of these compounds is required.

## Data Availability

The data used to support the findings of this study are available from the corresponding author upon request.
